# Point-of-care mobile digital microscopy and deep learning for the detection of soil-transmitted helminths and *Schistosoma haematobium*

**DOI:** 10.1080/16549716.2017.1337325

**Published:** 2017-08-25

**Authors:** Oscar Holmström, Nina Linder, Billy Ngasala, Andreas Mårtensson, Ewert Linder, Mikael Lundin, Hannu Moilanen, Antti Suutala, Vinod Diwan, Johan Lundin

**Affiliations:** ^a^ Institute for Molecular Medicine Finland (FIMM), University of Helsinki, Helsinki, Finland; ^b^ Department of Women’s and Children’s Health, International Maternal and Child Health (IMCH), Uppsala University, Uppsala, Sweden; ^c^ Department of Medical Entomology and Parasitology, School of Public Health, Muhimbili University of Health and Allied Sciences, Dar es Salaam, Tanzania; ^d^ Center of Microscopy and Nanotechnology, University of Oulu, Oulu, Finland; ^e^ Department of Public Health Sciences, Karolinska Institutet, Stockholm, Sweden

**Keywords:** mHealth for Improved Access and Equity in Health Care, Neglected tropical diseases, helminth, point-of-care, computer vision, global health

## Abstract

**Background:** Microscopy remains the gold standard in the diagnosis of neglected tropical diseases. As resource limited, rural areas often lack laboratory equipment and trained personnel, new diagnostic techniques are needed. Low-cost, point-of-care imaging devices show potential in the diagnosis of these diseases. Novel, digital image analysis algorithms can be utilized to automate sample analysis.

**Objective:** Evaluation of the imaging performance of a miniature digital microscopy scanner for the diagnosis of soil-transmitted helminths and *Schistosoma haematobium*, and training of a deep learning-based image analysis algorithm for automated detection of soil-transmitted helminths in the captured images.

**Methods:** A total of 13 iodine-stained stool samples containing *Ascaris lumbricoides, Trichuris trichiura* and hookworm eggs and 4 urine samples containing *Schistosoma haematobium* were digitized using a reference whole slide-scanner and the mobile microscopy scanner. Parasites in the images were identified by visual examination and by analysis with a deep learning-based image analysis algorithm in the stool samples. Results were compared between the digital and visual analysis of the images showing helminth eggs.

**Results:** Parasite identification by visual analysis of digital slides captured with the mobile microscope was feasible for all analyzed parasites. Although the spatial resolution of the reference slide-scanner is higher, the resolution of the mobile microscope is sufficient for reliable identification and classification of all parasites studied. Digital image analysis of stool sample images captured with the mobile microscope showed high sensitivity for detection of all helminths studied (range of sensitivity = 83.3–100%) in the test set (n = 217) of manually labeled helminth eggs.

**Conclusions:** In this proof-of-concept study, the imaging performance of a mobile, digital microscope was sufficient for visual detection of soil-transmitted helminths and *Schistosoma haematobium*. Furthermore, we show that deep learning-based image analysis can be utilized for the automated detection and classification of helminths in the captured images.

## Background

The neglected tropical diseases (NTD) are a group of disabling, chronic diseases that prevail in tropical and subtropical, resource-constrained areas with poor sanitation [1]. A majority of these diseases are caused by parasitic worms, of which the most common species affect more than 500 million people in sub-Saharan Africa [2,3]. The vast majority of NTDs here are caused by the soil-transmitted helminths *Ascaris lumbricoides, Trichuris trichiura* and hookworms, and the trematode *Schistosoma haematobium*. Together, these poverty-related infections result in over 415,000 annual deaths and the loss of 43.5 million daily-adjusted life years [4]. Compared with other age groups, preschool, school-aged and adolescent children tend to harbor higher numbers of parasites, resulting in impaired growth and development, diminished physical fitness and decreased neurocognitive abilities [5]. Hookworms and schistosomiasis also represent a significant cause of maternal morbidity and pregnancy complications [6,7].

Reliable, sensitive and easy-to-use diagnostic tests are crucial in control, surveillance and assessment of treatment efficacy of these diseases. Standard diagnosis of soil-transmitted helminth infections relies on microscopic examination of stool samples [8], typically prepared with the Kato-Katz method [9], whereas diagnosis of *S. haematobium* relies on microscopy of urine samples, prepared with various techniques of filtering and sedimentation [10]. As the vast majority of these NTDs occur in rural areas where high-quality microscopy services often are lacking [11], the need for novel diagnostic techniques is abundant [12]. Furthermore, the manual microscopic examination for the detection of helminths in a traditional stool sample is time-consuming, requiring on average 8–10 min for a skilled microscopist to reliably evaluate a sample [13].

During recent years, mass-production of cheap components for the consumer electronics market has enabled the construction of low-cost, portable imaging devices utilizing plastic lenses and components, mainly used in smartphone camera systems. Studies suggest that the diagnosis of helminth infections and schistosomiasis with this type of devices is technically feasible [14–18]. Recent studies have also introduced novel diagnostic approaches with innovative image-capturing techniques for applications at the point-of-care. Examples of these techniques include utilizing ball lenses placed on top of camera sensors for increased magnification and spatial resolution, or placing microscopy specimens directly on the surface of the sensor chip, which completely eliminates the need for optical lenses. By utilizing advanced computational holographic techniques, such as diffraction analysis and super-resolution by the pixel shift technique, even higher resolution imaging is achievable [19–24]. Often the trade-off for a higher spatial resolution is a decreased field of view, which is the case for example with external ball lenses. The majority of these devices support the digitization of relatively small sample areas at a time, typically one microscope field of view. In clinical applications, this can be cumbersome, as the microscopic examination of samples for parasites (e.g. helminths or schistosomes) typically requires examination of significantly larger areas, especially with samples showing low levels of infection or when assessing infection rate. Benefits of digital microscopy compared to light microscopy include real-time data sharing for remote diagnosis and consultations to facilitate faster diagnosis [25,26], and the possibility of utilizing digital image analysis to aid in sample analysis [27].

In this proof-of-concept study we evaluated the imaging performance of a low-cost, small-sized, cloud-connected, digital microscope for the diagnosis of the most common soil-transmitted helminth infections (*A. lumbricoides, T. trichiura* and hookworm) as well as *S. haematobium*. The device utilizes an external motor unit, which enables the digitization of larger sample areas, much in the same manner as a conventional whole slide-scanner [28]. Here, we evaluate the image quality of the captured images both visually, and by utilizing a deep learning-based computer-vision algorithm, operating on a remote server, to automatically analyze the stool samples and identify helminth eggs.

## Methods

### Parasite specimens

The stool samples for the experiments in this study were prepared in laboratory conditions from anonymous, previously collected fecal samples. The specimens were concentrated with ethyl acetate and fixed with formalin prior to storage. In total, three stool samples were acquired – one stool sample containing hookworm eggs, one containing *A. lumbricoides* and one containing a mixture of *T. trichiura* and *A. lumbricoides*. Furthermore, one sample consisting of unfiltered, unstained urine containing *S. haematobium* was acquired. The samples used here originated from collections of specimens collected for educational purposes and quality assurance, and were not collected specifically for this study. The stool samples were obtained from the Department of Microbiology and Immunology, University of Helsinki, Finland and the urine sample from the diagnostic parasitology laboratory of the Swedish Institute for Communicable Diseases Control (‘Panel för Cystor & Maskägg’, SMI, Solna, Sweden).

For the glass slides, we measured equal parts of stool sample and iodine staining solution. To fixate the sample on the microscope glass, we prepared an acrylamide solution from 40% acrylamide, phosphate buffer solution (PBS), tetramethylethylenediamine (TEMED) and ammonium persulfate. The stool sample was mixed with equal parts acrylamide solution, after which 20 µl droplets of the corresponding solution were placed on glass slides and covered with cover slips. Regarding the *S. haematobium* samples, we measured equal parts of the urine sample and the acrylamide solution described previously, and placed 20 µl droplets of the corresponding solution on the glass slides, which were covered with cover slips. To prevent drying and crystallization of the samples, the edges of the cover slips were coated with mounting media (‘Eukitt®’, ORSAtec GmbH, Augsburg, Germany). After the samples were prepared, they were immediately examined visually by a researcher (OH) using a light microscope with 40× magnification to confirm the presence of parasites. Samples not showing parasites when examined microscopically were discarded, and samples showing parasites were used for digitization. In total, we prepared 30 fixated stool samples and 10 urine samples. After exclusion of the samples lacking parasites, a total of nine glass slides showing *A. lumbricoides*, two of which also contained *T. trichiura*, five slides showing hookworm eggs and four slides showing *S. haematobium* parasites were further analyzed.

### Digital microscope and slide digitization

**Figure 1.**
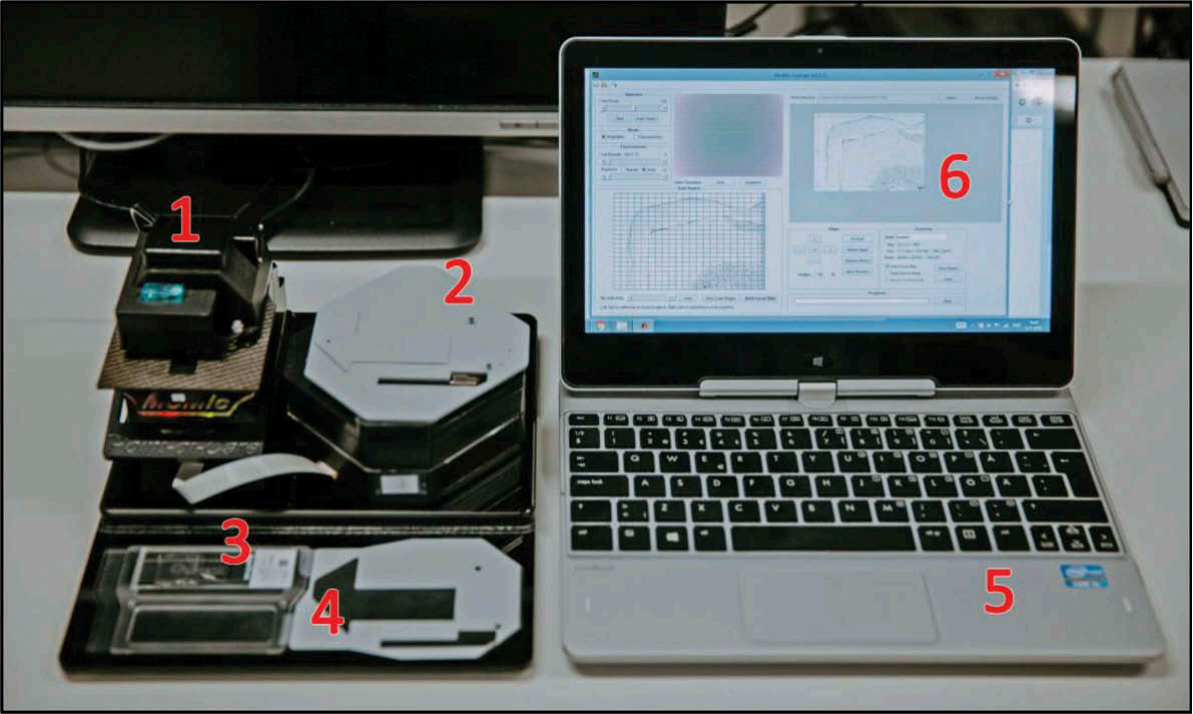
MoMic digital microscope scanner (1) with external motor unit attached (2). The microscope glass (3) is placed in the slide holder (4), which is placed in the microscope and navigated from the motor unit. The device is connected to and operated from a laptop computer (5) running software (6) for operation of the device.

The digital microscope used for the experiments in this article, is a small-sized, lightweight, cloud-connected digital microscope [29] (Figure 1). The microscope device used within this study is constructed from mass-produced, inexpensive components typically utilized in smartphone cameras. The total cost of materials for the construction of the prototype of the device is approximately that of a mid-range smartphone. The light source of the microscope is a white light-emitting diode (LED) for brightfield imaging, with a retractable band pass filter and an ultraviolet LED to support fluorescent imaging. The camera module (CM6787-O500BA-E, TRULY Optoelectronics Ltd., Hong Kong) of the microscope has a 5 megapixel complementary metal oxide semiconductor (CMOS) sensor with a maximum image resolution of 2595 × 1944 pixels. The plastic ¼ʺ lens used in the setup has an effective focal length of 3.37 mm. The microscope field of view is roughly 0.98 × 0.74 mm^2^ and the pixel size approximately 0.38 μm/px. This renders a spatial resolution of 1.23 μm, comparable to that of a traditional light microscope using a 10× objective with a numerical aperture of 0.25 (resolution approximately 1.10 μm). Coarse focus can be adjusted manually using a mechanical focus lever, and the built-in auto focus-routine is used for adjustment of fine focus. The microscope is connected, powered and operated through a universal serial bus (USB) from a laptop or tablet computer running a custom software program written in the matrix laboratory (MATLAB, MathWorks Inc, Natick, MA) to control the device and capture the images. Features of the software include a live stream from the camera, and control of brightness, focus, exposure and resolution (VGA [640 × 480] or QSXGA [2595 × 1944]). Basic image processing techniques, such as background correction and frame averaging, are used to optimize the image quality. Rough adjustment of the glass slide can be performed manually, and more precise adjustment is possible by utilizing the external motor unit. The motor unit is also used when digitizing areas larger than a single field of view to automatically navigate the sample while the device captures multiple images from different locations of the samples. Acquired images (representing single fields of view) are saved on the local storage of the computer and uploaded to an image processing and management platform (WebMicroscope, Fimmic Oy, Helsinki, Finland) running on a cloud server (University of Helsinki, Meilahti Campus Library Terkko, Helsinki, Finland). The image management server is password protected and secured by SSL (Secure Socket Layer) technology and located in a locked area at the Meilahti Campus (University of Helsinki, Finland). After the scanning process, the captured images are stitched to a larger digital slide. We used the commercially available software Microsoft Image Composite Editor (Microsoft Computational Photography Research Group, Microsoft Inc., Redmond, WA) for the creation of the digital whole-slides. The generated virtual slides were saved in the Tagged Image File Format (TIFF), and further compressed to a wavelet file format (Enhanced Compressed Wavelet [ECW], ER Mapper, Intergraph, Atlanta, GA) with a compression ratio of 1:9 to reduce file size, before uploading to the whole-slide image management server for remote access, e.g. by using a web browser. As shown in earlier work, this amount of compression preserves sufficient spatial detail to not alter results significantly [30].

**Figure 2.**
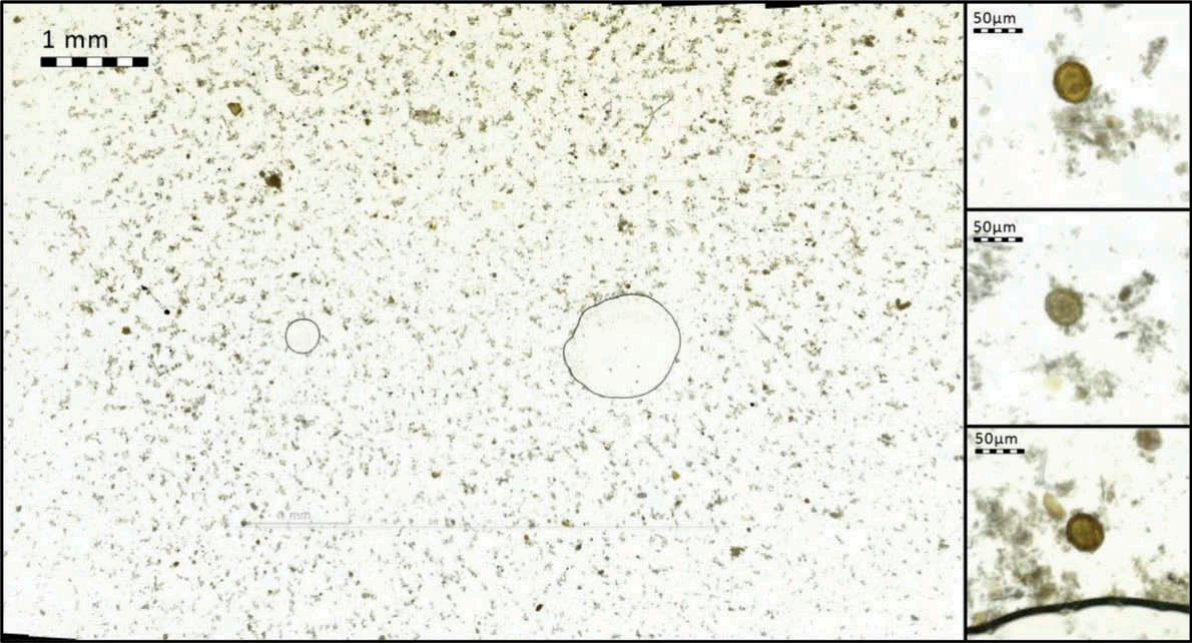
Region of glass slide captured with the mobile microscope. Enlarged areas (right) showing *A. lumbricoides* eggs in sample (magnified images showing native, full resolution of captured images at 100% digital magnification).

Scanning of the slides was performed immediately after preparation to prevent drying and crystallization of the acrylamide gel. Representative rectangular regions from the center of the cover glass were manually selected for digitization by visual examination. Since sample quality was not constant between slides (e.g. presence of air bubbles, staining artifacts and amount as well as distribution of parasites), the areas scanned with the mobile microscope varied, and on average measured 537 fields of view per sample (approximately 389.3 mm^2^) (Figure 2).

### Creation of reference digital slides

For reference purposes and to benchmark imaging performance, the samples were also digitized using a high-end, automated whole slide-scanner (Pannoramic P250, 3DHistech Ltd., Budapest, Hungary). This conventional whole slide-scanner uses a 20× objective (NA 0.8) equipped with a three-CCD (charge-coupled device) digital camera with a pixel resolution of 0.22 µm. The acquired images were compressed with a compression ratio of 1:9 to a wavelet file format and uploaded to the whole-slide management server, using the configurations previously described.

### Image analysis

For the digital image analysis of the samples, we used a commercially available image analysis software platform (WebMicroscope, Fimmic Oy, Helsinki, Finland). The software utilizes deep learning-based machine-learning algorithms to create software models for computer vision applications, i.e. analysis and classification of image features. The software supports creation of both supervised and unsupervised image analysis algorithms. Training of the algorithms was done by labeling a set of images on an object level. Images were annotated by indicating the center point of the object of interest. Based on the labeled areas the software then generates a deep learning model for locating candidate areas of interest, i.e. helminth eggs. The candidate areas were then assigned to different classes representing the different parasite species. The software also performs automatic perturbation of the training images, i.e. creating synthetic variations of images in the training data. Methods of perturbation include rotation, scaling, flipping and adjustment of contrast and luminance in the images.

The training set used for the creation of the algorithms consisted of 218 randomly selected images of the stool samples, representing single fields of view captured with the mobile microscope, manually confirmed to contain visible helminth eggs. The model for the identification of the parasites consists of two sequential algorithms. The first algorithm analyzed the whole image for helminth egg candidates, by comparing image features with learned parameters of the objects of interest. This algorithm was trained to identify any type of helminth egg, based on the manually annotated (location, size) regions of interest. The regions of interest detected by the first algorithm were then fed forward to a second classifier trained to identify the different helminth species. This classifier analyzed exclusively the candidate regions of interest (200 × 200 pixels, approx. 76 × 76 µm^2^) identified by the first algorithm, and based on probability classified these into one of four categories: *A. lumbricoides, T. trichiura*, hookworm or other (e.g. artifacts). The results of the analysis and classification were then exported from the software as a single panel showing the total number of fields of view analyzed, results from manual scoring and results from computer analysis. The actual regions of interest were also exported into the report, as well as an assigned value of probability of the candidate areas representing one of the four classes. The software calculated this value, with a probability percentage (0–100%) for each class, corresponding to a total sum of 100%. All color images (RGB) were converted into grayscale for the training and analysis.

### Statistical analysis

For the results of the digital image analysis, we calculated sensitivity for helminth egg detection on an object level as the percentage of true positives (TP) divided by true positives and false negatives (FN). Positive predictive value (PPV) on an object level was obtained as the percentage of true positives divided by true positives and false positives (FP).

## Results

**Figure 3.**
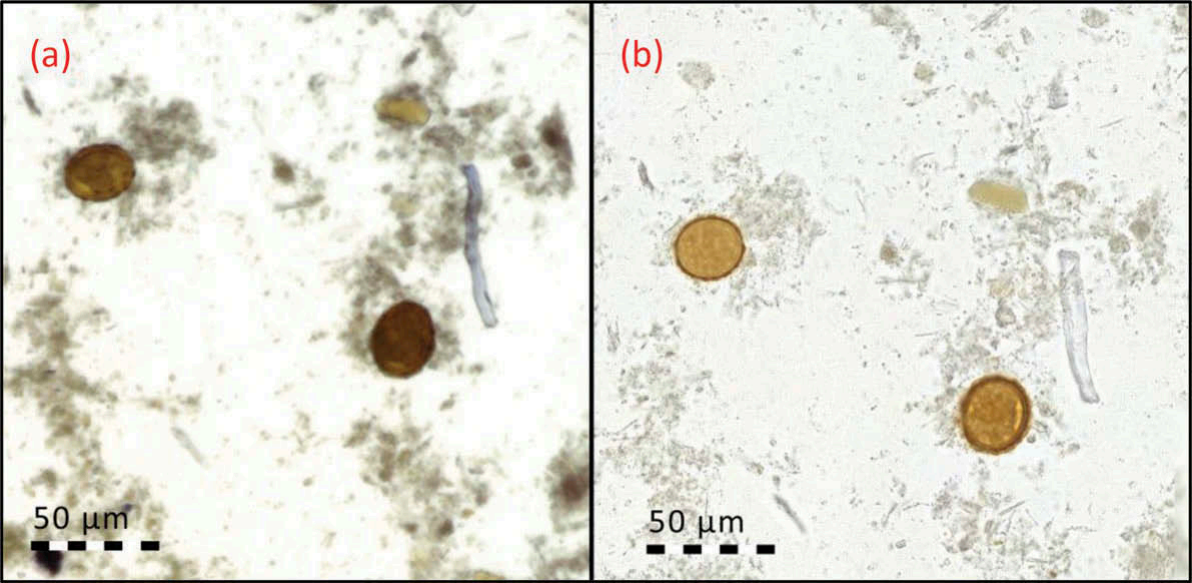
Stool sample showing *A. lumbricoides* eggs, digitized with (a) mobile microscope, and (b) reference slide-scanner.

**Figure 4.**
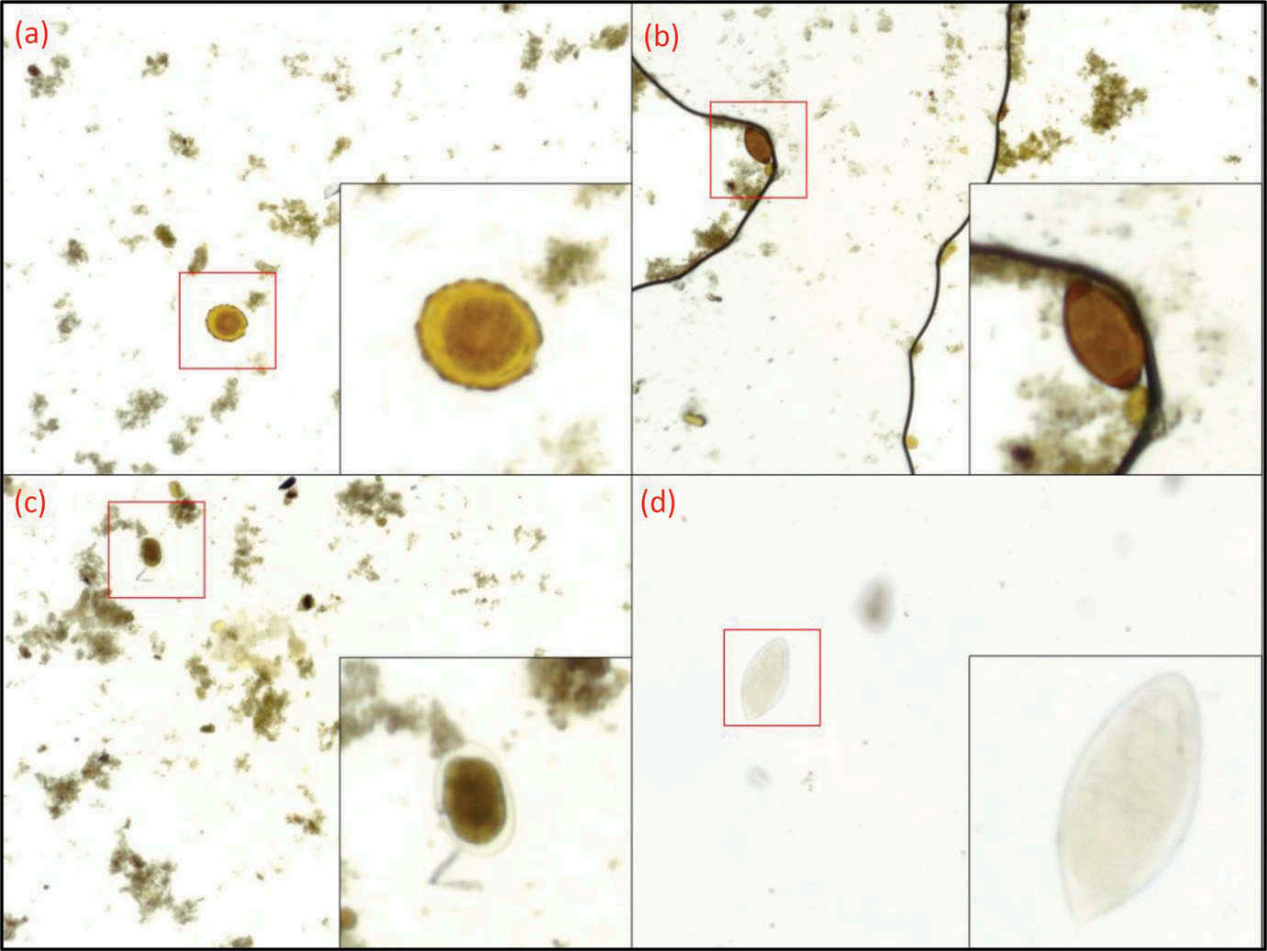
Captured images with the mobile microscopes with visible parasites. Enlarged areas showing the parasites at 300% digital zoom. (a) *A. lumbricoides*, (b) *T. trichiura*, (c) hookworm, (d) *S. haematobium*.

After digitization, the digital samples were visually examined on a computer monitor. For both the images acquired from the reference slide-scanner and the mobile microscope, the spatial resolution was sufficient for parasite eggs to be clearly distinguishable by visual examination (Figure 3). The spatial resolution of the reference slide-scanner was higher (i.e. parasites could be resolved in greater detail), but all species of parasites studied could be clearly resolved in the images acquired with both devices (Figure 4). We did not encounter samples where the parasites could be resolved with the reference slide-scanner but not the mobile microscope when comparing the digital slides.

### Identification of helminths in stool samples by computer vision

We compared the results from the digital image analysis of the images from the mobile microscope to the manual labeling of individual helminth eggs in the images (microscopic fields of view). This labeling was performed by two of the researchers and the labels where both observers (OH and JL) agreed both on location and on helminth species were used for the analysis.

For the digital image analysis all images that contained helminth eggs were pooled. The total number of images captured with the mobile microscope from all samples was 7385. Of these images, 410 were classified as positive (containing observable helminth eggs) by manual classification. In these images, the total number of labeled helminth eggs was 434, of which 390 were labeled as *A. lumbricoides*, 12 as *T. trichiura* and 32 as hookworm eggs.

For training of the algorithm, we used 50% of the labeled images (n = 205) containing a total of 217 parasite eggs (*A. lumbricoides*: 195, *T. trichiura*: 6, hookworm: 16). The other half of the images (n = 205) was used as a test set to evaluate the accuracy of the trained algorithm. The sensitivity of the algorithm for the detection of *A. lumbricoides* on an object level in the test set was 100%. The algorithm correctly classified all 195 labeled eggs in the test set as positive. The absolute number of FP *A. lumbricoides* eggs was 13 and FN 0, yielding an overall PPV of 93.7% on an object level. For *T. trichiura* the sensitivity obtained was 83.3%, the number of FP was 0 and FN 1, resulting in a PPV of 100% on an object level. The sensitivity for detection of hookworm eggs was 93.8%, the amount of FP 7 and FN 1, resulting in a PPV of 69.6% on an object level.

## Discussion

In this proof-of-concept study, the imaging performance of an inexpensive, portable digital microscope constructed from cellphone camera components and low-cost microelectronics was sufficient to reliably resolve the most common helminth eggs in stool and *S. haematobium* in urine samples visually in digitized slides. We show how the device can be used for the automatic digitization of larger sample areas (hundreds of microscopic fields of view) that is typically needed if a digital microscope is to be used for diagnostic purposes. Furthermore, we demonstrate how a deep learning-based image analysis algorithm can be developed for automatic helminth egg detection in the digitized samples. We achieved a relatively high sensitivity for parasite detection (83.3–100%) on an object level in the small test set of analyzed images, even though the number of samples available for training of the algorithm was limited. The sensitivity of the detection of the specific helminths correlated with the amount of training data (annotated images) available, and thus suggests that a larger number of images in the training set could possibly increase the sensitivity further. When compared to visual annotation of the captured images, the majority of cases differing from the manual labeling represented false positives (n = 20), i.e. objects in the images were incorrectly classified as helminth eggs. Overall, the number of labeled objects missed by the algorithm was clearly lower (n = 2). It is, however, important to note that the current study only presents results on an object level and in a subset of fields of view that contained helminth eggs. The number of FP is likely to increase in relation to the number of images analyzed.

**Figure 5.**
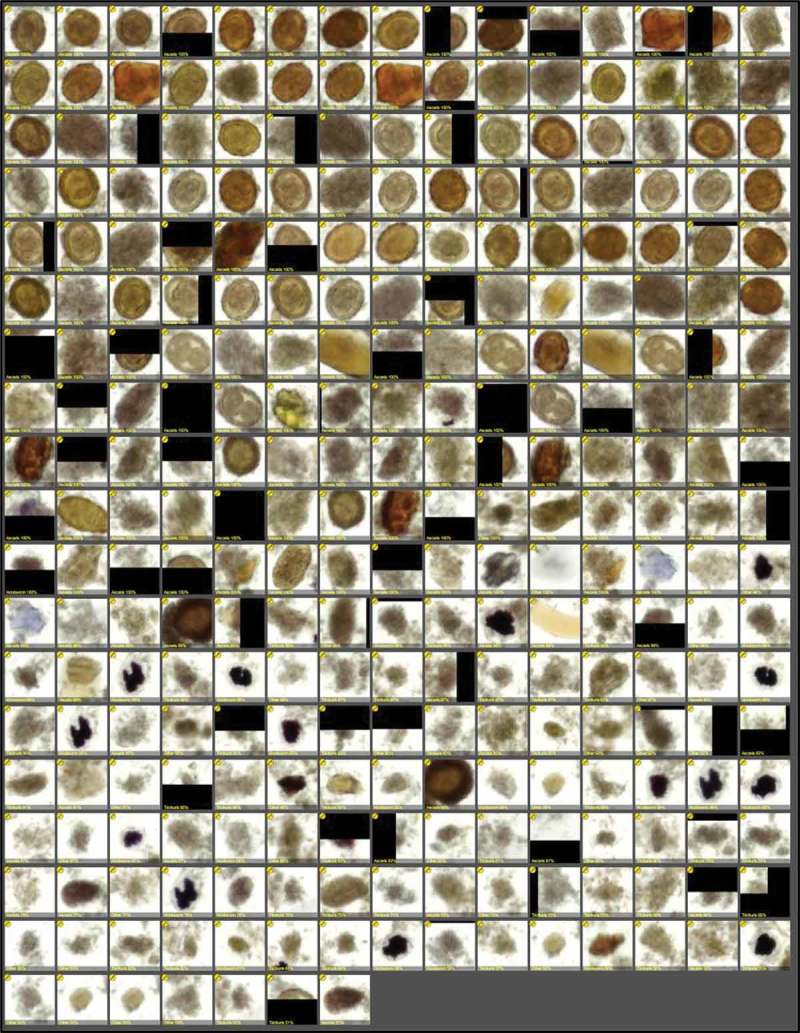
Results of digital image analysis of fixated stool sample (*A. lumbricoides* infection), showing detected regions of interest of fixated stool sample, sorted by certainty of representing parasite (descending order, from most likely candidates to least likely), as detected by the image analysis software.

A solution to tackle FP algorithmic detection is to include a manual review of the results from the digital image analysis. As shown in Figure 5, the exportation of the detected regions of interest (parasite candidates) from the software into a single panel, grouped from most likely to represent a parasite to least likely, allows the user to quickly review the most significant areas of the sample and, for example, confirm parasitic infection. By streamlining sample analysis in this way, this method of computer-assisted diagnosis could potentially result in faster sample analysis time by allowing the human end-user to review samples significantly faster and confirm the presence of parasites, while also reducing the number of FP detected by the software. By still leaving the final diagnosis to be confirmed by a human expert, faster adoption into clinical practice could potentially be allowed.

Different software solutions have been proposed for the automated identification and quantification of parasites, such as soil-transmitted helminths, in digitized samples with various levels of reported sensitivity and specificity [31,32]. Deep learning-based solutions to this type of pattern recognition tasks represent the state of the art in machine learning, and have recently attained significant attention due to the high performance of such algorithms in various image classification tasks [33–35]. This was the main reason for selecting this type of image analysis for this study.

Our study adds to the findings of previous studies by showing that identification of *A. lumbricoides, T. trichiura*, hookworms and *S. haematobium* is feasible at the point-of-care with a low-cost, digital microscope and by demonstrating how deep learning-based digital image analysis can be utilized to facilitate sample analysis and potentially reduce turnover time for sample evaluation. Compared to conventional digital slide scanners, which typically cost from 30,000 to 180,000 euros, this type of device should be possible to manufacture at a significantly lower cost (for approximately the price of a modern smartphone), and thus might be easier to implement, especially in resource-limited environments [36]. The main limitation of this study is the sparse number of samples analyzed. Because of this, we have made no attempt to objectively compare the sensitivity for parasite detection to standard reference methods (i.e. examination by light microscope), nor have we attempted to evaluate the performance in assessing overall level of infection in the samples. The limited amount of training images available for the image analysis algorithm, due to the low amount of samples, likely represents another limiting factor. We hypothesize that a larger training set would result in a significantly more robust algorithm with higher sensitivity. However, the results show that a relatively high sensitivity for parasite detection can be achieved even with a limited amount of training data. As we needed to immobilize the samples to be able to digitize them with both the reference slide-scanner and the mobile microscope, the samples were prepared using a different technique compared to traditional methods. Due to this, the results cannot be directly applied to samples prepared with other methods, such as conventional Kato-Katz smears.

Important features of future mobile, cloud-connected digital microscopy devices are sufficient imaging performance for rapid and accurate diagnosis, user-friendliness and cost-efficiency. Instruments should preferably support high-throughput, high-sensitivity screening of large populations with a rapid turnover time, which is especially important when evaluating changes in levels of infection in populations. This is essential for example when evaluating treatment efficacy in mass drug administration programs and assessing changes in infection prevalence on a population level [37]. As the performance and availability of global data networks are improving rapidly, digital samples can now be uploaded almost instantaneously from remote locations to cloud servers for remote consultation and computer-assisted diagnosis performed at a distance. As a complement to manual sample evaluation, this could potentially improve access to diagnosis in resource-constrained areas, decrease time for sample analysis and increase diagnostic accuracy. However, before decisions on implementation in clinical practice can be taken, clinical validation of this novel technology is needed. When planning and developing strategies for deployment, other factors such as development of training programs for technicians operating the device, clinical guidelines for usage and regular maintenance procedures need to be taken into consideration. Wider-scale implementation of novel technologies furthermore requires a coordinated effort from researchers, funders and policy-makers to ensure sustained access and availability [38].

## Conclusions

This proof-of-concept study shows that the imaging performance of an inexpensive, digital microscope is sufficient to reliably resolve the most common helminth eggs (*A. lumbricoides, T. trichiura* and hookworms) in fixated stool samples and *S. haematobium* in urine samples. Furthermore, we demonstrate how a deep learning-based image analysis algorithm can be utilized for automated detection of helminth eggs in stool samples. These findings add to previous work showing that inexpensive, digital imaging devices can be utilized for diagnosis of these diseases at the point-of-care in rural areas. Further validations of these techniques are required before implementations in clinical environments.

## Paper context

Neglected tropical diseases affect more than a billion people globally, mainly in rural areas lacking resources for diagnostics. Portable, digital microscopes have been suggested as potential novel diagnostic tools. Here we demonstrate how a low-cost, miniature digital microscope scanner, supported by artificial intelligence-based sample analysis, can be used for field diagnostics of common parasitic diseases. Parasite identification with this technology is feasible, although further clinical validation is needed before wider implementation.
